# Dual Biological Functions of a Cytoprotective Effect and Apoptosis Induction by Bioavailable Marine Carotenoid Fucoxanthinol through Modulation of the Nrf2 Activation in RAW264.7 Macrophage Cells

**DOI:** 10.3390/md15100305

**Published:** 2017-10-09

**Authors:** Junsei Taira, Miki Sonamoto, Masatsugu Uehara

**Affiliations:** Department Bioresource Technology, Okinawa National College of Technology, 905 Henoko, Nago-city, Okinawa Prefecture 905-2192, Japan; sonamoto.mb@om.asahi-kasei.co.jp (M.S.); koe2jikpqvxy8cbnuvri0xyzlgd1nv@ezweb.ne.jp (M.U.)

**Keywords:** marine carotenoid, fucoxanthinol, Nrf2, apoptosis, ESR, peroxy radical

## Abstract

In this study, the function of fucoxanthinol (FxOH) as a bioavailable marine carotenoid together with the pre-metabolite, fucoxanthin (Fx), was examined through the Nrf2-ARE pathway. The antioxidant activity in the low concentration range of the compounds (1–4 μM) with a peroxyl radical scavenging capacity was proved by the ORAC (Oxygen Radical Absorbance Capacity) method and an ESR study. Similar concentrations of the compound also activated the Nrf2-ARE signaling with the Nrf2 translocation into the nuclear, then the expression of the antioxidant protein HO-1 increased. On the other hand, the high concentrations of both compounds (>10 μM) induced apoptosis with caspase 3/7 activation during suppression of the anti-apoptotic proteins, such as Bcl-_X_L and phosphorous Akt (pAkt). The Nrf2 expression was then activated in the nuclear, indicating that the Nrf2 plays a significant role in the cytoprotective effect against the toxicity of the compounds. These results indicated that the compounds have the dual functions of a cytoprotective effect and the apoptosis induction dependent on the treated concentrations through the Nrf2 activation. In addition, the results of all the assays involved in our previous studies suggested that the metabolite FxOH having a higher activity than the Fx, will be a bioavailable compound in biological systems.

## 1. Introduction

The seaweeds having functional marine carotenoids are expected to be food materials for preventing the oxidative stress related to various diseases. A major marine carotenoid, fucoxanthin (Fx), was found in the edible seaweeds such as *Undaria pinnatifida*, *Sargassum fulvellum,* and *Hijikia fusiformis*, and their physiological functions were elucidated, such as their anti-carcinogenic effects [1,2,3], apoptosis activation [4], and anti-inflammatory effects [5]. The marine carotenoids, such as Fx and its metabolites, fucoxanthinol (FxOH), and halocynthiaxanthin, also demonstrated that they have an antioxidant activity due to the scavenging of reactive oxygen species (ROS), such as O_2_^−^ and •OH [6]. In our recent study, the anti-inflammatory effect of FxOH together with Fx was assessed based on the NO production in LPS stimulated inflammatory macrophage cells, and they suppressed the NO production with inhibition of the iNOS mRNA expression and the reactive nitrogen species (RNS) scavenging activity [7]. 

In response to the oxidative stress due to the ROS and RNS, the cells attempt induction of the antioxidant enzymes as the first line of defense. The Nrf2 (Nuclear factor-erythroid-2-related factor) -ARE (antioxidant response element) signaling is the cell survival response to avoid cell damage by the excess production of the ROS and RNS or electrophiles. The dissociation of the Nrf2 from the Kelch-like ECH associated protein 1 (Keap1) by electrophiles and oxidative stress as a consequence of the Keap1 cysteine thiol modification induces DNA sequences located in the promoter and enhancer regions as ARE-mediated phase II detoxifying/antioxidant enzymes, such as NAD(P)H:-quinone oxidoreductase-1 (NQO1), glutathione S-transferase (GST), thioredoxin, and heme oxygenase-1 (HO-1) [8]. Certain dietary chemopreventive agents target the Keap1 by oxidizing or chemically modifying its specific cysteine thiols, which can induce the ARE-mediated expression [9,10,11]. Dietary Fx was deacetylated to FxOH in the intestinal tract by lipase and esterase from the pancreas or in the intestinal cells, and incorporated as FxOH from the digestive tract into the blood circulation system in mammals, thus FxOH is considered to be a bioavailable compound in biological systems [4,7,12]. However, the cellular defense function of the metabolite FxOH against oxidative stress involving apoptosis induction through the Nrf2–ARE-mediated antioxidant enzymes has not yet been clarified. Therefore, this study placed an aim to elucidate the functions of FxOH together with Fx on the Nrf2-ARE signaling. As a result, it is elucidated that the compounds provide the dual functions of the cytoprotective effect due to the activation of the antioxidant protein and the apoptosis induction dependent on the treated concentrations through the Nrf2 activation. In addition, the results of all the assays involved in our current studies suggested that the metabolite FxOH, will be a real bioavailable compound in biological systems.

## 2. Results

### 2.1. Antioxidant Capacity by Peroxyl Radical Scavenging

**Figure 1:**
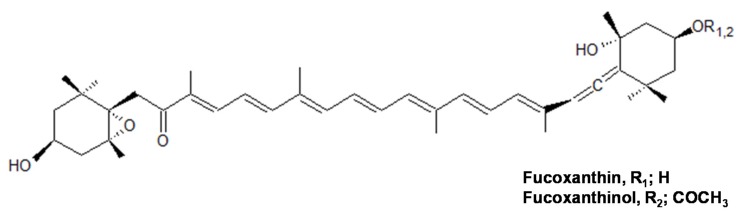
Chemical structures of marine carotenoids, fucoxanthin (Fx) and its metabolite fucoxanthinol (FxOH). FxOH is produced by the deacetylation of Fx in the intestinal tract with lipase and esterase.

**Figure 2:**
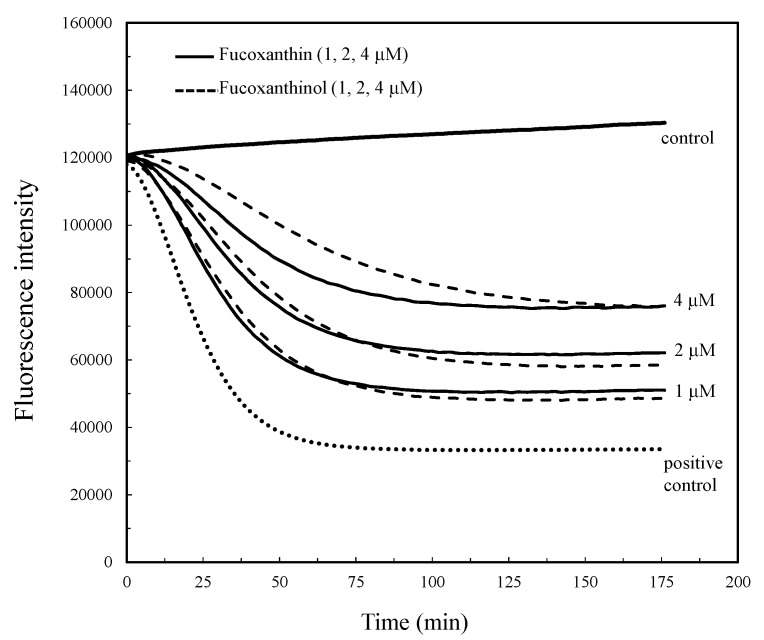
Antioxidant capacity of marine carotenoids, fucoxanthin (Fx) and its metabolite fucoxanthinol (FxOH). The antioxidant capacity of the Fx and FxOH in the range of 1–4 μM, was evaluated by a peroxyl radical generating system using the ORAC (Oxygen Radical Absorbance Capacity) method as shown in the text.

Peroxyl radicals (ROO•) were generated by the thermal decomposition of AAPH, and the decay of the fluorescence probe during the reaction with the ROO• was monitored with or without the compounds (Figure 1). The compounds indicated an antioxidant activity with the ROO• scavenging capacity in the low concentration range of the compounds (1–4 μM) (Figure 2). The antioxidant activity of the compounds was indicated as the Trolox equivalent activity (TEA). The linear relationship, *Y*_net AUC_ (AUC_sample_ − AUC_positive control_) = 27.005 × (μM Trolox concentration) + 5.810, between the Trolox concentrations and netAUC then indicated a correlation coefficient of *r*^2^ = 0.998, which was used to determine the mol-TEA/mol-compound of the test compounds. The antioxidant capacities of FxOH and Fx were 0.73 ± 0.03 and 0.72 ± 0.03 mol-TEA/mol-compound, respectively.

**Figure 3:**
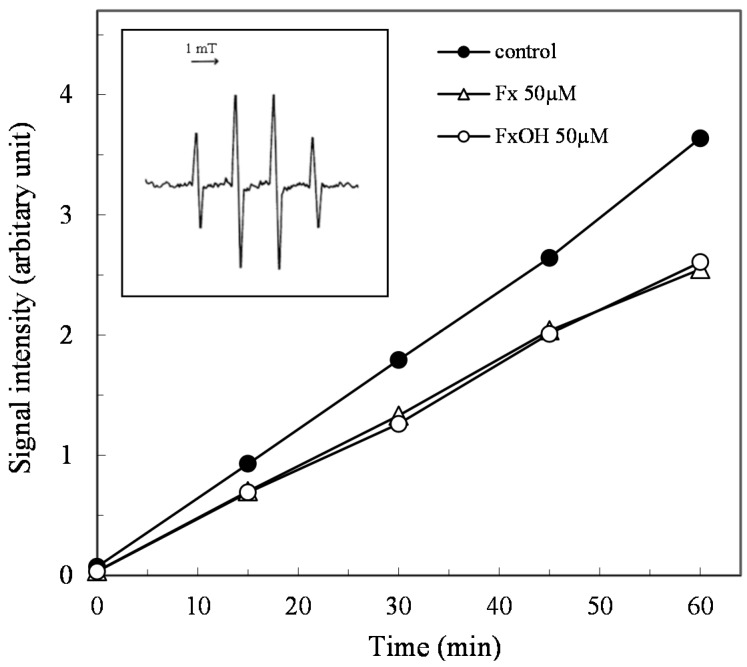
Peroxy radical scavenging action of marine carotenoids, fucoxanthin (Fx) and its metabolite fucoxanthinol (FxOH). The peroxy radical (ROO•) scavenging action due to the marine carotenoids (50 μM), the Fx and its metabolite FxOH was confirmed by the ESR study, as described in the text. The ESR signal of the DMPO-ROO• spin adduct inserted in the figure was monitored with or without (control) compound during the incubation time for 180 min.

In addition, the ROO• scavenging activity was evaluated by the ESR study using the spin trapping method. The ROO• formed by the thermal decomposition of AAPH was detected by DMPO as a spin trap reagent. The DMPO-ROO• spin adduct was reduced in the presence of compounds during the incubation time, indicating that the compounds have a potential ROO• scavenging activity (Figure 3).

### 2.2. Cytotoxicity of the Compounds

**Figure 4:**
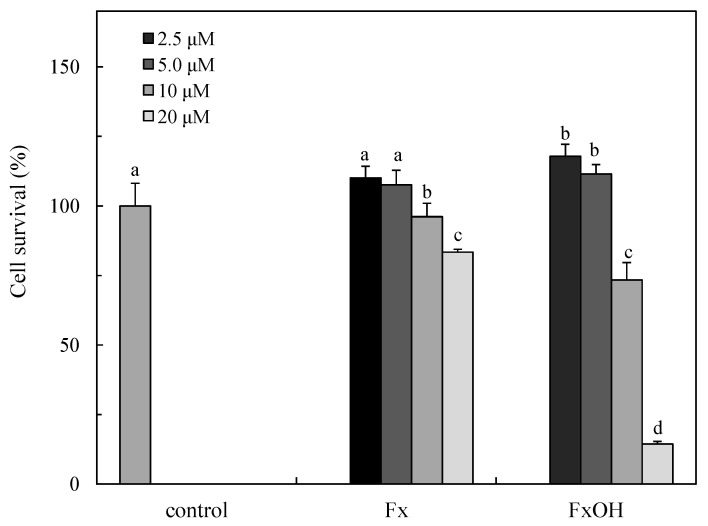
Cytotoxicity of the marine carotenoids, fucoxanthin (Fx) and its metabolite fucoxanthinol (FxOH) in RAW 264.7 macrophage cells. The cytotoxicity of the compounds was determined by an MTT assay and indicated as survival rate (%) vs. the control cells without compounds. Data were expressed as mean (*n* = 4) ± SD. Analysis of data was carried out using ANOVA and Tukey’s method for multiple comparisons. A significance *p <* 0.05 between two bars with the different alphabetical letters was considered statistically significant.

The cytotoxicity in the presence of the compounds is indicated in Figure 4. The treatment of Fx (IC_50_s, 49.06 μM) at 20 μM indicated cytotoxicity, while similar concentrations of FxOH (IC_50_s, 12.65 μM) led to complete cell death (Figure 4). 

### 2.3. Nrf2-ARE Signaling

**Figure 5:**
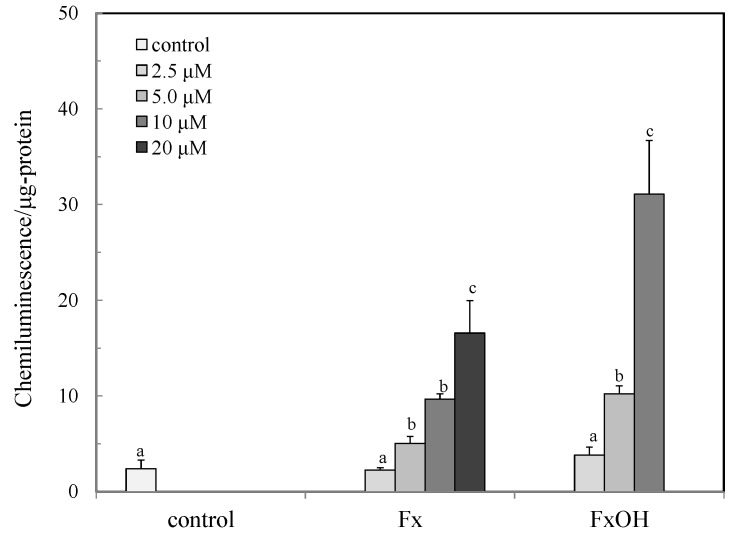
Activation of Nrf2-ARE signaling due to marine carotenoids, fucoxanthin (Fx) and its metabolite fucoxanthinol (FxOH) in RAW 264.7 macrophage cells. The activation of Nrf2-ARE signaling due to marine carotenoids, the Fx (2.5–20 μM) and FxOH (2.5–10 μM) was evaluated by the reporter assay, as described in the text. Data were expressed as mean (*n* = 4) ± SD. Analysis of data was carried out using ANOVA and Tukey’s method for multiple comparisons. Significance *p <* 0.05 between two bars with the different alphabetical letters was considered statistically significant.

The activations of the Nrf2-ARE signaling in the presence of the compounds are shown in Figure 5. The signal was activated in a dose dependent manner in the range of 2.5–10 μM for FxOH and 2.5–20 μM for Fx. The activation of FxOH was 2–3 fold higher than those of the Fx treated cells. The signal was then activated even under the cell death concentration (>10 μM) of both compounds (Figure 5). 

### 2.4. Nuclear Nrf2 Expression 

**Figure 6:**
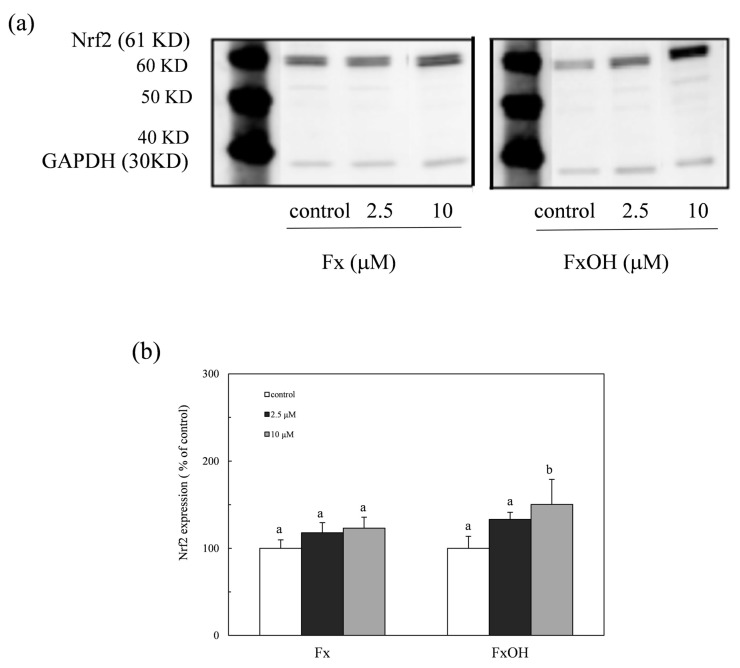
Expression of the nuclear Nrf2 protein due to marine carotenoids, fucoxanthin (Fx) and its metabolite fucoxanthinol (FxOH) in RAW 264.7 macrophage cells. The Nrf2 translocated into the nuclear in the presence of compounds (2.5 and 10 μM). (**a**) Western blot analysis of the Nrf2 protein in the presence of compounds. (**b**) Densitometry analysis of the expression of Nrf2 protein (**a**). Data were expressed as mean (*n* = 3) ± SD. Analysis of data was carried out using ANOVA and Tukey’s method for multiple comparison. Significance *p <* 0.05 between two bars with the different alphabetical letters was considered statistically significant.

The expression of the nuclear Nrf2 protein was determined by western blot analysis, as shown in Figure 6a,b. The Nrf2 translocated into the nuclear in the presence of compounds during the Nrf2-ARE signaling. The protein expression in the nuclear due to the FxOH treatment cells was also higher than that of Fx, which was similar to the result of the Nrf2-ARE signaling activation (Figure 6).

### 2.5. HO-1 Expression

**Figure 7:**
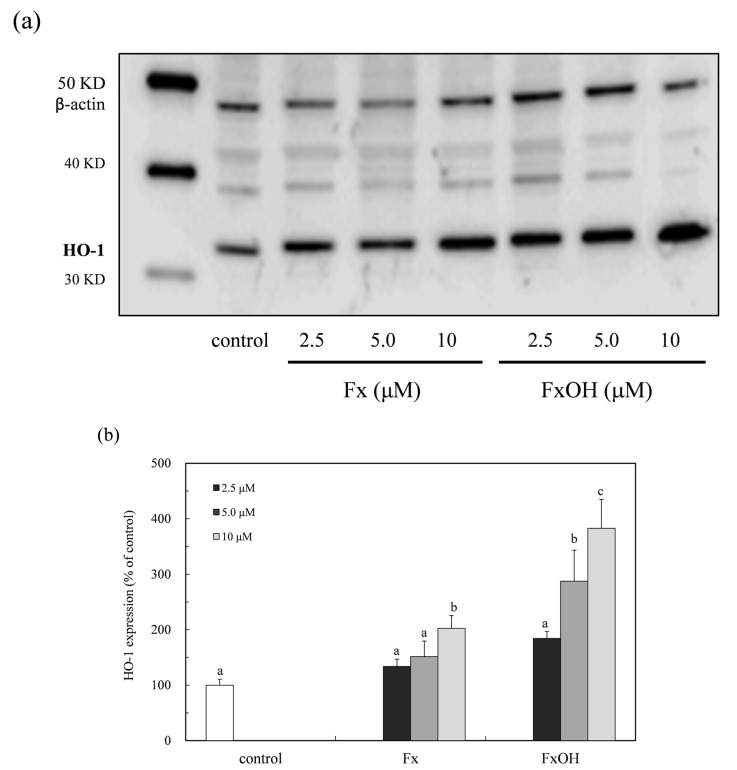
Expression of HO-1 expression due to marine carotenoid, fucoxanthin (Fx) and its metabolite fucoxanthinol (FxOH) in RAW 264.7 macrophage cells. The HO-1 protein expression due to marine carotenoids (2.5–10 μM), the Fx and its metabolite FxOH effect on the Nrf2-ARE signaling in the cells was examined. (**a**) Western blot analysis of the HO-1 protein in the presence of compounds. (**b**) Densitometry analysis of the expression of HO-1 protein. Data were expressed as mean (*n* = 3) ± SD. Analysis of data was carried out using ANOVA and Tukey’s method for multiple comparisons. A significance *p <* 0.05 between two bars with the different alphabetical letters was considered statistically significant.

The HO-1 protein expression in the presence of compounds was determined by a western blot analysis (Figure 7a). The protein expression increased in the low concentration range (2.5–10 μM) of the compounds (Figure 7b). This result indicated that the compounds have an antioxidant activity due to the HO-1 protein induction with nuclear Nrf2 activation. In addition, the HO-1 protein expression due to the FxOH treatment cells was two-fold higher than that of the Fx treatment.

### 2.6. Apoptotic Cell Induction

**Figure 8:**
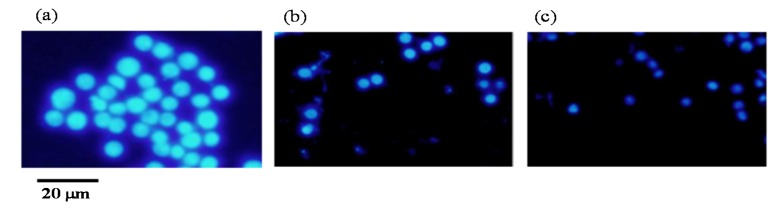
Apoptotic cell induction due to marine carotenoid, fucoxanthin (Fx) and its metabolite fucoxanthinol (FxOH) in RAW 264.7 macrophage cells. The apoptotic cells were stained using a Hoechst 33342 dye. The cells were treated with (**b**) Fx (10 μM) and (**c**) FxOH (10 μM) or without (**a**) the compounds (control). The apoptotic cells with the nuclear condensation were detected due to the compound treatment.

The cells were treated with compounds (b) Fx and (c) FxOH or without ((a) control) and then the apoptotic cells were examined by staining using Hoechst 33342 dye. As shown in Figure 8, the cells with the nuclear condensation were detected in the presence of the compounds indicating apoptotic cell death.

### 2.7. Caspase Activity

**Figure 9:**
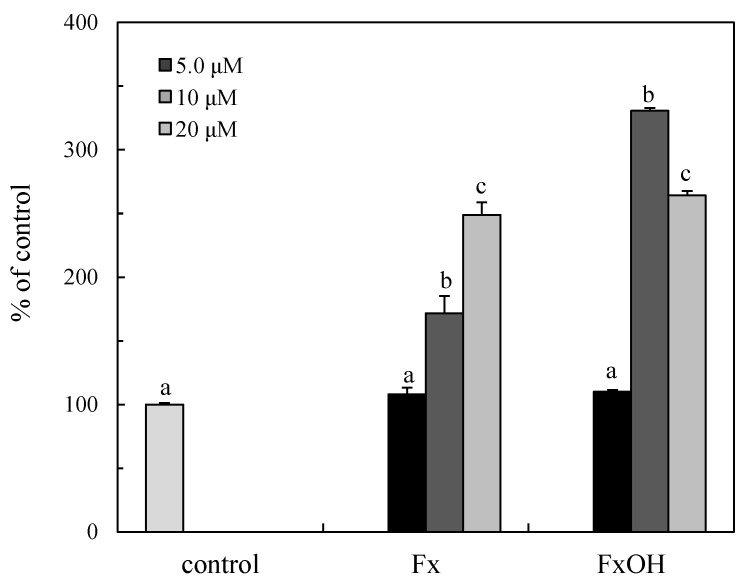
Caspase 3/7 activity of marine carotenoids, fucoxanthin (Fx), and its metabolite fucoxanthinol (FxOH) in RAW 264.7 macrophage cells. The caspase 3/7 activity of the Fx and its FxOH was expressed as percent compared to the control cells without compound. Data were expressed as mean (*n* = 3) ± SD. Analysis of data was carried out using ANOVA and Tukey’s method for multiple comparisons. A significance *p <* 0.05 between two bars with the different alphabetical letters was considered statistically significant.

**Figure 10:**
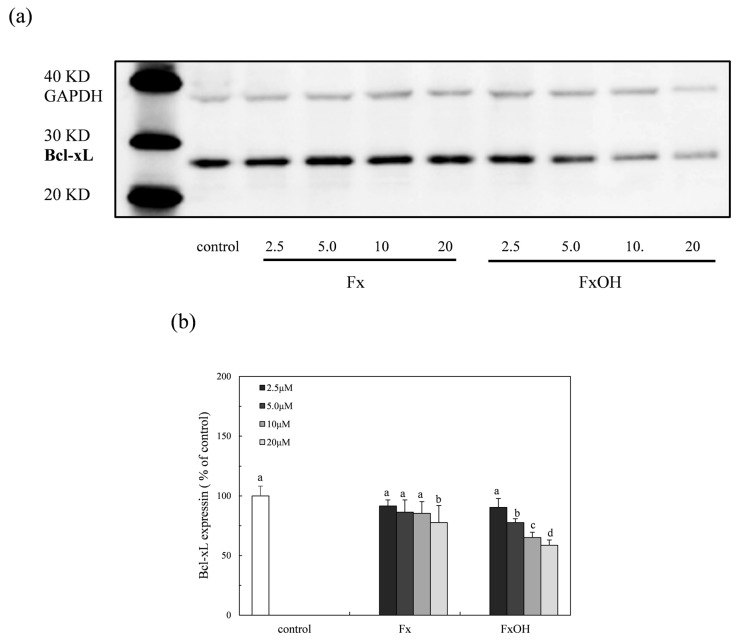
Suppression of the Bcl-xL protein expression due to marine carotenoids, fucoxanthin (Fx) and its metabolite fucoxanthinol (FxOH) in RAW 264.7 macrophage cells. The Bcl-xL protein expression with Fx and FxOH in the range of 2.5–20 μM on the Nrf2-ARE signaling in cells was examined. (**a**) Western blot analysis of the Bcl-xL protein in the presence of compounds. (**b**) Densitometry analysis of the expression of Bcl-xL protein. Data were expressed as mean (*n* = 3) ± SD. Analysis of data was carried out using ANOVA and Tukey’s method for multiple comparisons. A significance *p <* 0.05 between two bars with the different alphabetical letters was considered statistically significant.

**Figure 11:**
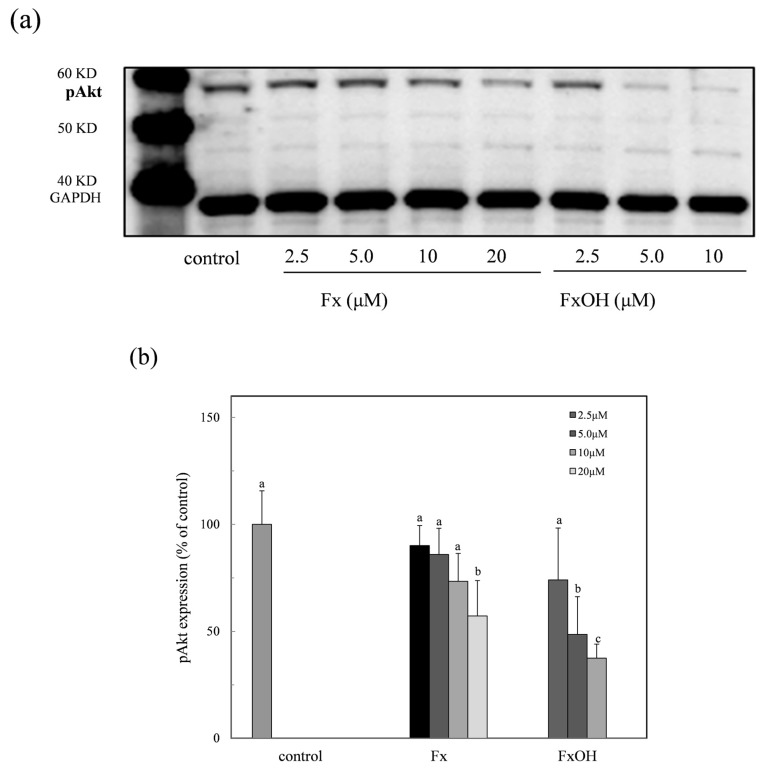
Suppression of the phospho-Akt (pAkt) expression in the presence of marine carotenoids, fucoxanthin (Fx) and its metabolite fucoxanthinol (FxOH) in RAW 264.7 macrophage cells. The pAkt protein expression due to the Fx and FxOH in the range of 2.5–20 μM on the Nrf2-ARE signaling in cells was examined. (**a**) Western blot analysis of the pAkt protein in the presence of compounds. (**b**) Densitometry analysis of the expression of pAkt protein. Data were expressed as mean (*n* = 3) ± SD. Analysis of data was carried out using ANOVA and Tukey’s method for multiple comparisons. A significance *p <* 0.05 between two bars with the different alphabetical letters was considered statistically significant.

Caspases are activated in the caspase cascade with apoptotic cell death. The caspase 3/7 activities of the downstream in the cascade were examined in the presence of the compounds. The caspase 3/7 was activated by the treatment compounds at > 10 μM (Figure 9), then similar concentrations of these compounds induced cell death with the induction of apoptosis. The caspase activity of FxOH at 20 μM is similar to that of 10 μM treatment that led to complete cell death with the suppression of antiapoptotic proteins, as indicated the following results (Figure 10 and Figure 11). The caspase activation of the FxOH treated cells was two-fold higher than that of Fx, which was similar to the cytotoxicity result of the compounds (Figure 4).

### 2.8. Bcl-xL Protein Expression

The Bcl-xL protein is an anti-apoptosis factor due to the suppression of cytochrome c release, which is a trigger in the caspase cascade. The expression of the Bcl-xL protein in the presence of compounds on the Nrf2-ARE signaling was examined. The protein expression was suppressed in the presence of compounds (Figure 10). Specifically, the inhibition of the FxOH treatment cells was higher than that of the Fx, which was similar to the result of the apoptotic cell induction with caspase 3/7 activation (Figure 8 and Figure 9). 

### 2.9. Phospho-Akt Protein Expression

The anti-apoptotic protein phospho-Akt (pAkt) through the PI3K-Akt signaling pathway was examined in the presence of the compounds. The expression of the pAkt with the apoptotic induction was inhibited in a dose dependent manner (Figure 11a). Particularly, the protein expression of the FxOH treated cells was clearly suppressed in the low concentration range of 2.5–12 μM (Figure 11b). This result was similar to that of the Bcl-xL expression (Figure 10). Thus, the inhibition of the anti-apoptotic proteins due to the compound treatment leads to apoptosis. 

## 3. Discussion

The ROS and RNS are widely recognized as being involved in the pathogenesis of various diseases and aging processes. The antioxidant activities of the marine carotenoids, such as Fx and its metabolite FxOH, are due to the radical scavenging activity against the ROS [6]. Additionally, this study examined the antioxidant capacity of the compounds for scavenging of the ROO• related to the cell membrane injury. The low concentrations of the compounds showed the antioxidant activity involved in the ROO• scavenging activity (Figure 2 and Figure 3). In our recent study, the compounds demonstrated an anti-inflammatory effect through suppression of the NO production and the RNS scavenging activity on the LPS-induced inflammatory macrophage cells [7]. Except for the chemical protection against oxidative stress involving the ROS and RNS, the cells attempted the regulation of the Nrf2-ARE signaling [8]. Upon the oxidative stress and electrophiles, the Nrf2 parts from Keap1 and translocates into the nucleus to induce the expression of HO-1 as an antioxidant protein [13]. The antioxidant capacity of the test compounds through the Nrf2-ARE signaling was examined, then the compounds activated the signaling, resulting in the elevation of the HO-1 protein (Figure 5 and Figure 7). A previous study reported the activation of the Nrf2-ARE signaling due to Fx, but the actual active compound has not been yet clarified [14]. Our study demonstrated that the metabolite FxOH has a higher activity than that of Fx that suggested the metabolite FxOH, will be a real bioavailable compound in biological systems. When the cysteine residue in keep1 is oxidized due to electrophiles, then the Nrf2 transfers to the nuclear and binds to the ARE region on the gene. The carbonyl function in the compounds may probably contribute to the oxidization of the silyl group in the Keep1, resulting in the accumulation of Nrf2 in the cytoplasm and then translocated into the nuclear. In addition, the intermediates produced from reaction with the ROS, for example, the reaction products with ROO•, may change to an electrophilic form (Figure 2 and Figure 3).

Previous studies reported that the marine carotenoids including neoxanthin and Fx induced apoptosis against cancer cells, such as lymphomas and prostate cells [4,15]. This study further clarified the apoptosis induction mechanism by Fx and its metabolite FxOH in normal cells. The compounds induced apoptosis with the caspase 3/7 activity downstream of the caspase cascade during the Nrf2-ARE signal activation (Figure 5, Figure 8, and Figure 9). The pAkt protein regulates apoptosis related proteins, such as Bcl 2, Bad, and Bcl-xL [16]. While the expressions of pAkt, including Bcl-xL, as anti-apoptotic proteins were suppressed, an induction of apoptosis resulted (Figure 8, Figure 10, and Figure 11). Specifically, the suppression of these proteins due to FxOH was remarkably higher than that of Fx, which also elevated the caspase activity (Figure 9). The Nrf2-ARE signaling in the presence of compounds was activated during the apoptosis induction. In additional experiments, when the cells treated with a higher concentration (20 μM) of FxOH, which completely induced cell death, then the nuclear Nrf2 translocation was also inhibited (data not shown). Therefore, the results indicated that the Nrf2 plays a significant role in the cytoprotective effect against toxicity of the compounds. A previous study reported that 4-ketopinonresinol isolated from adlay induced the Akt phosphorylation and subsequently phosphorylated Nrf2 expressed in nuclear through the Nrf2/ARE signaling related to the cytoprotective proteins, such as HO-1, aldo-keto reductases, and glutathione synthetic enzyme [17]. The compounds inhibited the expression of pAkt, which may induce apoptosis due to the down regulation of the PI3K/Akt signaling involved in the Nrf2 phosphorylation. As another role of Nrf2 in cancer cells, it is known to directly or indirectly facilitate the metabolic pathways and the cell proliferation through the PI3K-Akt signaling [18,19,20]. This study first suggested that the compounds induced apoptosis due to the suppression of the Nr2 activity except for the Nrf2-Are signaling pathway.

Based on these results, the compounds have the dual functions of a cytoprotective effect and the apoptosis induction through the Nrf2 activation. In addition, the results of all the assays involved in our previous studies suggested that the metabolite FxOH having a higher activity than the Fx, will be a bioavailable compound in biological systems [7].

## 4. Materials and Methods 

### 4.1. Materials 

Caspase-Glo 3/7, pGL4.37 [luc/ARE/Hygro], and FuGENE 6 plasmid were obtained from the Promega Corporation (Madison, WI, USA). The products of anti-Nrf2 (Santa Cruz Biotechnology, Inc., Dallas, TX, USA), anti-HO-1 (StressMarq Biosciences Inc., Victoria, BC, Canada), anti-Bcl-xL (BioVision, Inc., Milpitas, CA, USA), anti-phospho Akt (Cell Signaling Technology Japan, Inc., Tokyo, Japan), and anti-β-actin (Wako Pure Chemical Industries, Ltd., Osaka, Japan) were used for detecting the protein expression. 3-(4,5-Dimethyl-2-thiazlyl)-2,5-diphenyltetrazolium bromide (MTT) and fluorescein were obtained from Nacalai Tesque, Inc. (Kyoto, Japan). 5, 5′-Dimethyl pyrolline N-oxide (DMPO) as a spin trap reagent was purchased from Dojindo Laboratories (Kumamoto, Japan). 2,2′-Aazobis (2-methylpropionamidine) dihydrochloride (AAPH) was purchased from Wako Pure Chemical Industries, Ltd. (Osaka, Japan). Fucoxanthin (Fx) from *Nemacystus decipiens* and its deacetylated, fucoxanthinol (FxOH) were obtained from South Product, Ltd. (Okinawa, Japan). 

### 4.2. Peroxyl Radical Scavenging Capacity

The antioxidant capacity of the compounds was evaluated by a peroxyl radical generating system as previously reported [12]. Briefly, the reaction mixture in the presence of AAPH (8 mM) and fluorescein (6.8 nM) as a fluorescence probe with or without the test compounds (1, 2, and 4 μM) in phosphate buffer (75 mM, pH 7.0) was incubated for 180 min at room temperature. The fluorescence decay due to the reaction with the peroxyl radicals was monitored using a microplate reader (Glomax Multi Detection System, Promega KK, Madison, WI, USA). The peroxyl radical scavenging capacity (%) was determined by an ORAC assay and it was calculated from the net integrated areas under the fluorescence decay curves (AUC) using the following equation [21]. 

Peroxyl radical scavenging capacity (%) = (AUC_sample_ − AUC_positive control_/AUC_negative control_ – AUC_positive control_) × 100

The positive control contained AAPH without the sample, while the negative control had no sample and AAPH. A standard curve was made from the linear relation between the netAUC and 0.25–2 μM Trolox concentrations, and the linear relationship, *Y*_net AUC_ (AUC_sample_ − AUC_positive control_) = *a* × (μM trolox concentration) + *b*, was used to assess the antioxidant capacity of the test compounds. The values were expressed as mol-Trolox equivalent activity/mol compound (mol-TEA/mol-compound). 

### 4.3. ESR Measurement

The peroxy radical scavenging action of the compounds was confirmed by an ESR study, as previously reported [22]. The reaction mixture of the compound (50 μM, 50% DMSO), AAPH (10 mM), and spin trap reagent, DMPO (452.5 mM) was prepared in PBS and incubated for 60 min at room temperature. An ESR measurement was performed by ESR spectroscopy (JES-FR30, JEOL) operating in the X-band with a modulation frequency of 100 kHz. The reaction mixture was transferred to a capillary (100 × 1.1 mm I.D., Drumnond Scientific Co., Broomall, PA, USA), which was placed in a quartz cell (270 mm long, 5 mm I.D., JEOL DATUM Ltd., Tokyo, Japan). The ESR spectra was measured at a 9.4 GHz resonant frequency under the following conditions: microwave power, 4 mW; modulation width, 0.1 mT; gain, 320; scan time, 1min; time constant, 0.1 s Manganese oxide was used as the internal standard. 

### 4.4. Cell Culture

RAW264.7 cells (mouse macrophages, American Type Culture Collection, Manassas, VA, USA) were cultured in DMEM medium (including 10% FBS, 100 U/mL penicillin and 100 μg/mL streptomycin) at 37 °C in a 5% CO_2_ atmosphere. 

### 4.5. Cell Viability 

The cell viability treatment with a test sample was examined by an MTT assay, as previously reported [23]. Briefly, MTT (0.05%) was added to each well after the culture and incubated for 3 h, and the suspension was carefully removed. The formazan reduced from an MTT was extracted with DMSO (100 μL) and measured as an index of the survival cells at 570 nm with the reference at 630 nm using a microplate reader (BIO-RAD Model 550, BIO-RAD, Hercules, CA, USA).

### 4.6. Reporter Assay of Nrf2-ARE Signaling

The RAW264.7 cells with or without the test sample were pre-cultured on a 12-well microplate (5 × 10^5^ cells/well) for 24 h the day before being transiently co-transfected in triplicate with the pGL4.37 [luc/ARE/Hygro] plasmid and using FuGENE 6 as the transfection reagent. After 24 h, the cells were washed twice in PBS and lysed in 100 μL lysis buffer. The lucyferase activity of the lysate cells (50 μL) was assayed using a luciferase substrate, and then the chemiluminescence (CL) was measured by a microplate reader (GLOMAX MULTI Detection system, Promega). The protein concentration was determined using a BCA protein assay kit (Thermo Fisher Scientific, Inc., Waltham, MA, USA).

### 4.7. Nuclear Extraction

Cells (2.0 × 10^6^ cells/mL) with or without the test samples at the final concentrations of 2.5 and 10 μM were pre-incubated for 24 h. After 24 h, the cells were washed twice in PBS and lysed in 100 μL lysis buffer. The nuclear in the cells was extracted by manual procedures using an extraction kit (NE-PER Nuclear and Cytoplasmic Extraction Reagent, Thermo Fisher Scientific K.K, Yokohama, Japan). Nrf2 protein detection using anti-Nrf2 was carried out by western blot analysis and the Nrf2 protein expression was expressed as a % of the untreated sample cells (control).

### 4.8. Apoptotic Cell Detection

The apoptotic cells were detected by Hoechst 33342 staining, as previously reported [14]. Briefly, pre-cultured cells (5.0 × 10^5^ cells/mL) were treated with the test compound (10 μM) for 24 h. After washing the cells with PBS, then incubated with Hoechst 33342 (10 μg/mL) for 30 min at room temperature. Following the washing of the cells, the apoptotic cells were detected using a fluorescence microscope (Olympus, IX71) with a filter at the excitation of 350 nm and an emission of 461 nm.

### 4.9. Caspase 3/7 Activation

Cells were seeded at 1.0 × 10^5^ cells in a 96-well plate. After the culture for 24 h, the test compounds were treated for 17 h. The caspase 3/7 activity was measured using the Caspase Glo kit (Promega, WI, USA) according to the manufacturer’s instructions. Cells were incubated with 100 μL of Caspase Glo reagent at room temperature for 60 min. Following the caspase cleavage, the substrate reacts with luciferase and releases luminescent light in the presence of ATP and oxygen. The luminescence of the reaction products was measured using a microplate reader (GLOMAX MULTI Detection system, Promega, WI, USA). 

### 4.10. Western Blot Analysis

The cells were washed with PBS, and then treated with the lysis buffer. The cellular lysates were centrifuged at 13,800 g for 5 min. The total cellular extracts were separated on SDS-polyacrylamide gels (4–12% SDS-polyacrylamide, Invitrogen, Waltham, MA,, USA) and transferred to a nitrocellulose membrane (iBlot Gel Transfer Mini, Invitrogen) using an iBlot Gel Transfer Device (Invitrogen). The protein detection was carried out using an immunodetection system (Invitrogen) with the antibodies.

### 4.11. Statistical Analysis

Data were expressed as mean ± SD. Analysis of data was carried out using one-way analyses of variance (ANOVA) and Tukeỳs method for multiple comparison. Significance *p <* 0.05 was considered statistically significant.

## 5. Conclusions

This study demonstrated that the Fx and its metabolite FxOH provide a cytoprotective effect due to the anti-oxidative activity involving the HO-1 protein activation through the activation of the Nrf2-ARE pathway in the low concentration range. The treatment with a high concentration treatment of cells induced apoptosis with caspase 3/7 activation during the suppression of anti-apoptotic proteins, such as Bcl-_X_L and pAkt. The nuclear Nrf2 expressed during apoptosis activation then indicated that the Nrf2 plays a significant role in cell survival against toxicity of the compounds. These results indicated that the compounds have the dual functions of a cytoprotective effect, and apoptosis induction activity that is dependent on the concentrations through the Nrf2 activation.
